# Association between third molar agenesis and dental maturity in Brazilian children

**DOI:** 10.1007/s12024-025-01126-0

**Published:** 2025-11-22

**Authors:** Giselle Santiago da Cunha Zanqueta, Allan Abuabara, Thais Vilalba Paniagua Machado do Nascimento, Maria Angélica Hueb de Menezes-Oliveira, Isabela Ribeiro Madalena, Christian Kirschneck, Cristiano Miranda de Araujo, Flares Baratto-Filho, Erika Calvano Küchler, César Penazzo Lepri

**Affiliations:** 1 Department of Biomaterials, University of Uberaba - UNIUBE, Uberaba, Minas Gerais Brazil; 2 Universidade da Região de Joinville (University of the Joinville Region – Univille), Joinville, Santa Catarina Brazil; 3 School of Dentistry, Tuiuti University of Paraná – UTP, Curitiba, Paraná Brazil; 4 Department of Orthodontics, Medical Faculty, University Hospital Bonn, Bonn, Germany; 5 Tuiuti University of Paraná – UTP, Curitiba, Paraná Brazil; 6 University of Uberaba – Uniube, Uberaba, Minas Gerais Brazil

**Keywords:** Anodontia, Age determination by teeth, Forensic dentistry, Molar, Third, Panoramic radiography

## Abstract

The aim of the current study is to investigate if third molar agenesis is associated with dental maturity variations in Brazilian children. Radiographs of children from 10 to 15 years old were examined and patients with 32 teeth were included in the control group and patients with at least one third molar agenesis were included in the agenesis group. Demirjian’s method was used to calculate dental age and evaluate dental maturity. Dental age was calculated using the Dental Age mobile app. The dental maturity was determined by calculating the difference between dental age and chronological age (DA-CA), in which positive, negative, and null values indicated advanced, delayed, or normal dental development, respectively. T test and ANOVA were used for comparisons. The Pearson correlation test was used to evaluate the relationship between chronological age and dental age (alpha = 5%). A total of 336 patients were included, 58 (17.3%) had at least one third molar agenesis. Third molar agenesis was associated with delayed dental maturity (*p* = 0.0294). Patients with 3 or 4 missing third molars had statistically significantly more delayed dental development compared with control patients (*p* = 0.0001) and patients with 1or 2 third molar agenesis (*p* = 0.0290). Individuals with third molar agenesis present delayed dental maturity. Our results also suggested that the number missing third molars are associated with the delayed maturity. Individuals with third molar agenesis showed delayed dental maturity, which was more pronounced in those missing three or four third molars. These findings suggest that the extent of agenesis is associated with the magnitude of maturational delay.

## Introduction

Dental agenesis is a congenital anomaly consisting of the absence of one or more teeth. It is among the most common developmental dental anomalies, with prevalence rates varying by population and diagnostic criteria [1]. Excluding third molars, dental agenesis affects approximately 5% of the population. Agenesis prevalence differs by continent and sex. The prevalence for both sexes in Europe is 5.5% (males 4.6%; females 6.3%) while in North American the prevalence is 3.9% (males 3.2%; females 4.6%). Mandibular second premolars and maxillary lateral incisors are the most frequently missing teeth [2]. When third molars are included in the analysis, the prevalence rises significantly, with studies indicating that third molar agenesis occurs in more than 20% of individuals worldwide. A systematic review and meta-analysis were performed including 92 studies, containing 63,314 subjects. The study observed the worldwide rate of agenesis was 22.63% (estimates ranged from 5.32% to 56.0%) [3].

Dental maturation refers to the process by which teeth develop and progress through specific stages of formation, including eruption, and root completion. It is often used to assess age in both clinical and forensic settings. Dental maturation typically follows a predictable sequence, although timing can vary due to genetic, environmental, and nutritional factors. The assessment of dental age is commonly based on the development of tooth crowns and roots using radiographic methods such as Demirjian et al. (1973) [4]. The Demirjian’s method is a classification that estimates a child's age based on the stage of development of their permanent teeth from the onset of calcification to the final mature form with closure of the root apex [4, 5].

Studies suggest that certain genes associated with the congenital absence of tooth germs (dental agenesis) may also be the cause of delayed dental development [6]. Previous studies investigated the association between tooth agenesis and dental maturity and some studies observed that patients with dental agenesis have delayed dental maturity compared to controls [6–10]. Although the connection between dental development and agenesis of different teeth types have been extensively reported, there is not enough evidence if patients with third molar agenesis present delayed dental development. Understanding what influences dental age estimation is crucial for several reasons. It helps to create more accurate and reliable age assessments by accounting for biological variations, which is vital for forensic identification and legal age determination in cases like immigration or criminal proceedings. In clinical settings, this knowledge aids dentists in precise treatment planning for children and can even help diagnose developmental issues. Ultimately, identifying these influencing factors drives the improvement of existing methods and the development of new, more robust techniques for age estimation. Therefore, the aim of the current study is to investigate if third molar agenesis is associated with dental maturity variations in Brazilian children.

## Materials and methods

### Study design and setting

This cross-sectional retrospective study was conducted using secondary data from digital panoramic radiographs obtained between 2018 and 2023. The images were provided by a university dental clinic located in Uberaba, Minas Gerais, in southeastern Brazil. Sampling was performed by convenience, selecting images from an existing database of panoramic radiographs originally acquired for dental treatment purposes. This study was reported following the Statement of Strengthening the Reporting of Observational Studies in Epidemiology (STROBE) [11]. Approval was obtained from the ethics committee of the Centre for Health Sciences at Uberaba University (Uberaba, Minas Gerais, Brazil) under protocol CAAE 69181023.7.0000.5145. The procedures used in this study adhere to the tenets of the Declaration of Helsinki.

### Participants

Orthopantomographs and clinical records of children of both sexes were screened and selected. Only patients from 10 to 15 years old were included following that design from Ferreira and Caldas (2024) [12]. All participants received dental care within the same clinical setting, which follows standardized treatment protocols and procedures. Patients with underlying syndromes, with congenital alterations including cleft lip and/or palate and oligodontia, were excluded. In the control group, only patients with 32 teeth were included.

### Sample size

The sample size was determined assuming a third molar agenesis frequency around 20% as previously estimated in a systematic review and meta-analysis [3], therefore, a case:control ratio of 1:4 was used for the calculation, with a power of 80% and an alpha of 5%. A mean difference between the groups of 0.4 and a standard deviation of 0.9 was assumed. The sample size calculation estimated 250 patients, a sample of 50 patients with third molar agenesis and 200 controls were necessary. Taking the inclusion and exclusion criteria into account, we screened 400 dental records, of which a total of 336 patients were included.

Third molar agenesis was diagnosed using orthopantomographs by evaluating the presence or absence of the third molars in all four quadrants of the jaws. Radiographs were carefully examined to identify whether the tooth germ, crown, or any developmental stages of the third molars were visible. If no evidence of third molar formation was observed in the expected anatomical region, the condition was classified as agenesis. To ensure diagnostic accuracy, evaluations were performed by a trained examiner under standardized conditions, with any ambiguous cases re-evaluated to confirm the findings.

### Dental age estimation

Orthopantomographs from patients' dental records were evaluated by two examiners to assess dental age (DA). Prior to evaluating the orthopantomographs, the examiners underwent a training and calibration process. This included a theoretical discussion of Demirjian’s method followed by a practical session analyzing 20 panoramic radiographs [4]. Each examiner evaluated the radiographs twice, with a one-week interval between assessments. The intra- and inter-rater reliability was assessed by comparing their ratings with those of an experienced reference examiner in Demirjian’s method. The intra- and inter-rater kappa coefficients were 0.90 and 0.82, respectively, indicating strong agreement.

The evaluation focused on the permanent left mandibular teeth (excluding the third molar), which were rated on a scale from A to H based on their stage of dental mineralization. If a permanent tooth on the left side of the mandible was missing, the corresponding tooth on the right side was assessed. Children with one or more bilaterally missing teeth were excluded from the study. DA was then calculated using the Dental Age mobile app [13], which converted the mineralization stage ratings of the seven assessed teeth into a dental age value based on the child's sex, using tables provided by Demirjian et al. (1973) [4]. The variation in dental age was determined by calculating the difference between dental and chronological ages (DA-CA). The mean of these differences (*bias*) indicates whether dental age is systematically overestimated (positive values) or underestimated (negative values), while the standard deviation (*imprecision*) reflects the variability of this estimate [14].

### Statistical analysis

For the statistical analysis, the case group was evaluated as presenting at least one anomaly, and also stratified according to the affected dental arch (maxilla and mandible) and according to severity (1 or 2 missing teeth and 3 or 4 missing teeth). GraphPad Prism 8.2 (Graph-Pad, San Diego, CA, USA) was used for the calculations. In line with the Central Limit Theorem, when sample sizes are sufficiently large (typically above 30–40 observations), the sampling distribution of the mean approximates a normal distribution regardless of the original distribution of the data, which supports the application of parametric tests in biomedical research [15, 16]. Therefore, the *t*-test was used for comparisons of means (bias) and standard deviations (imprecision) of dental maturity (DA—CA) between groups. ANOVA and the post hoc Tukey test was used to compare the groups according to the number of missing third teeth. The Pearson correlation test was used to evaluate the strength of the relationship between chronological age and dental age and also to evaluate the e strength and direction of the correlation between dental age and number of missing third molars. The established alpha was 5%.

## Results

**Fig. 1:**
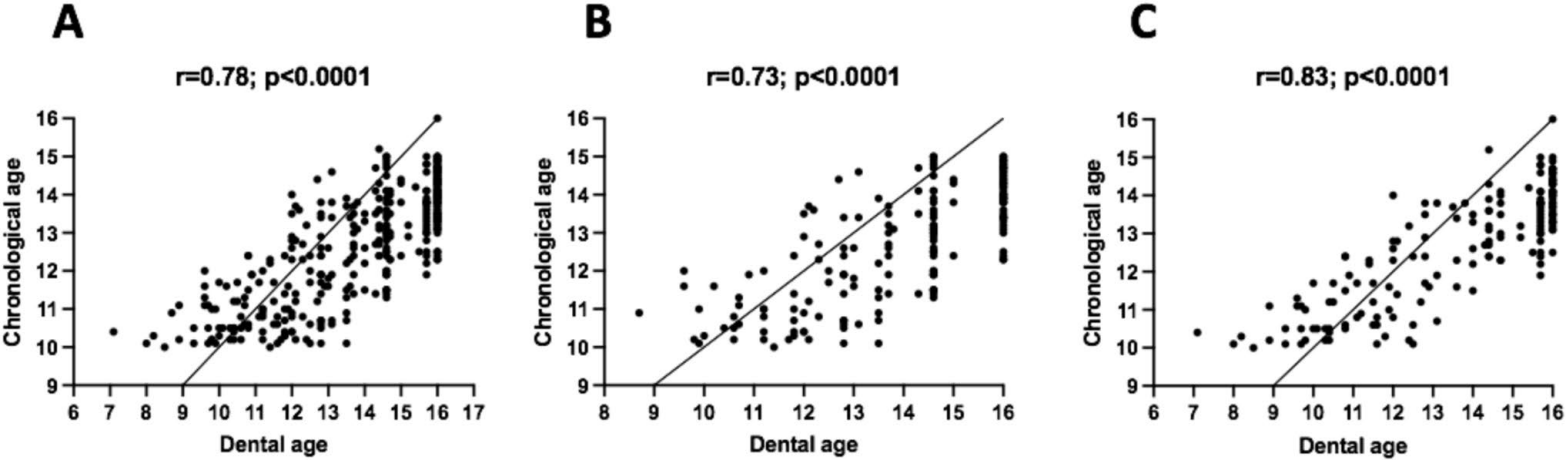
Correlation between chronological age and dental age. A-total sample; B- Girls; C-Boys

Of the total 336 patients included, 169 (50.3%) were girls and 167 (49.7%) were boys. The sample characteristics are presented in the Table 1. Sex was not associated with third molar agenesis (*p* = 0.901). The method overestimated the age for both, with a bias of 1.14 years and imprecision of 1.31 for controls, and a bias of 0.66 years and imprecision of 1.47 for the third molar agenesis group. A strong correlation between dental age and chronological age was observed for both sexes (Fig. 1).

**Table 1:** Characteristics of the studied population

Characteristics	Control	Third molar agenesis
Sample size (n)	278	58
Sex distribution	140 girls and 138 boys	29 girls and 29 boys
Chronological age range (min –max)	10.0; 15.9	10.0; 15.0
Chronological age (mean SD)	12.74 (1.44)	12.02 (1.59)
Dental age range (min –max)	8.0; 16.0	7.10; 16.0
Dental age range (mean SD)	13.96 (2.00)	12.69 (2.49)
Dental maturity (Dental age – Chronological age) range	2.40; 3.80	−3.30; 3.40
Dental maturity (Dental age – Chronological age) mean (SD)	1.14 (1.31)	0.66 (1.47)

Third molar agenesis was associated with delayed dental maturity (*p* = 0.0294). The analysis stratified according to the dental arch also demonstrated a statistical association (*p* = 0.0024 for maxillary third molar agenesis and *p* = 0.0104 for mandibular third molar agenesis). These results are shown in Table 2.

**Table 2:** Dental maturity (Dental age – Chronological age) according to the groups

Groups	*n*	Bias (mean)	Imprecision (SD)	*P*-value
Control	278	1.14	1.31	Reference
Third molar agenesis	58	0.66	1.47	0.0294*
Maxillary third molar agenesis	39	0.49	1.49	0.0024*
Mandibular third molar agenesis	33	0.44	1.59	0.0104*

The control group served as the reference group for the analysis. All *p*-values represent comparisons between each test group and the control. T test was used. *means statistical difference (*p* < 0.05)

**Fig. 2:**
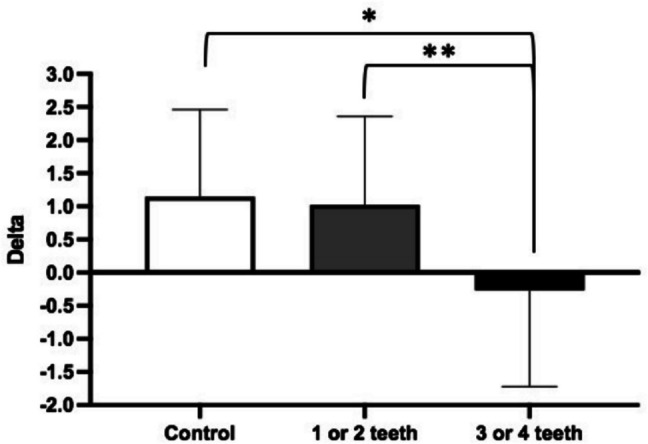
Dental maturity according to the number of missing third molars and controls. *p = 0.0001; **p = 0.0290

The number of missing third molars ranged from 1 to 4 the mean number of missing teeth was 2.17 ± 1.09. The mean number of missing teeth in females was 2.21 ± 0.99, while in males the mean number of missing teeth was 2.14 ± 1.19. Seventeen patients had only one third molar agenesis, 25 patients had 2 third molar agenesis, 3 patients had 3 third molar agenesis, and 13 patients had all 4 third molar agenesis. The bias for the control group was 1.14 (imprecision = 1.31), the bias for the group with 1 or 2 third molar agenesis was 1.02 (imprecision = 1.33); while the bias for the group with 3 or 4 third molars agenesis was −0.26 (imprecision = 0.36). Patients with 3 or 4 missing third molars had a statistically significantly more delayed dental development compared with control patients and patients with 1or 2 third molar agenesis (Fig. 2).

## Discussion

Dental maturity is a highly effective indicator of chronological age, often surpassing skeletal methods for assessing an individual's physical development. This makes it a valuable tool for estimating age in forensic and archaeological investigations. However, to improve the accuracy of these estimations, it is crucial to consider influencing factors [12]. Therefore, to investigate is third molar agenesis is a significant one that is highly relevant in dental and forensic science. In the current study, we investigated Brazilian children and teenagers in order to evaluate if third molar agenesis influences dental maturity and found some interesting results.

Our results support that studies aiming to estimate the chronological age of children and teenager should take into consideration third molar agenesis and the number of missing teeth. This is highly important due to the high prevalence of third molars congenitally missing. Third molar agenesis is more prevalent than other teeth agenesis, affecting approximately one quarter of the population [17]. It is one of the most prevalent developmental anomalies in humans. Recent studies have reinforced the clinical and diagnostic relevance of this dental anomaly, not only as an isolated manifestation but also as a potential marker of alterations in the chronology of dental development [5, 18, 19].

It is important to emphasize that some important aspects should be taken into consideration regarding third molar agenesis. Third molars are the last teeth to begin and complete their mineralization [20]. Therefore, the diagnosis of third molar agenesis cannot be performed in young children. Our study only included children older than 10 years old following a similar previous study [12]. Uzamiş et al. (2000) reported that the earliest age for maxillary third molar radiolucent crypt was eight years; and for mandibular third molars crypts, they could be seen radiographically as early as seven years [21]. Sarnat et al. (2003) reported that the first appearance of a radiolucent crypt was at age 8.7 in the mandible and 9.3 in the maxilla with no significant differences in the development of the third molar between boys and girls [22].

In the current study we used Demirjian's method to analyze dental maturity. According to the recent systematic review by Ferrilo et al. (2024), dental maturation analysis according to Demirjian's method can be considered reliable [23]. The assessment of dental age through tooth development analysis is a tool commonly used in clinical, forensic, and epidemiological research. The method proposed by Demirjian et al. (1973) remains one of the most widely adopted worldwide due to its practicality, reproducibility, and reliance on morphological criteria of dental mineralization [4]. The method is based on the assessment of the seven left mandibular teeth, assigning developmental stages (from A to H) and corresponding scores to estimate the dental age of children and teenagers between 3.5 and 16 years old.

Dental development is considered a reliable indicator of somatic maturation and is less influenced by environmental factors than other markers, such as bone age [20]. As observed in our study with third molar agenesis, previous studies have shown delay in dental development in individuals with tooth agenesis [24]. Some studies previously investigated the association between dental agenesis (excluding third molars) and dental maturity using Demirjian's method. The study by León-Rubio et al. (2022) in Spanish children with dental agenesis showed that patients with dental agenesis have delayed dental formation [6]. Additionally, they reported that in approximately 15% of cases, the contralateral homologous tooth to the missing one exhibited a developmental stage below the expected level for age, further supporting the hypothesis that agenesis is not merely a localized phenomenon, but rather a phenotypic expression of systemic alterations in dental development patterns. Kan et al. (2010), in Australian children, also observed a delayed dental development in the dental agenesis group [25]. Tunç et al. (2011) studied Turkish children and reported that children with mild-to-moderate dental agenesis had delayed dental development [26]. Ruiz-Mealin et al. (2012) investigated a population from the United Kingdom andconcluded that the development of permanent teeth in children with dental agenesis is delayed when compared with a matched control group [8]. Although our study focused on third molar agenesis, we observed similar results suggesting that dental agenesis, regardless of the type of missing teeth, is associated with delayed dental development/maturity.

Another interesting aspect observed in our study is that patients with the most severe phenotype—absence of all four third molars—exhibited the greatest delay in dental development. This finding suggests a connection between the severity of third molar agenesis and the dental maturation process, which should be taken into consideration in future analyses.

This study has some limitations. Potential variables associated with dental agenesis and dental development — including genetic factors, ethnic background, nutritional status, systemic health, and socioeconomic status — were not evaluated, as the primary objective was to assess the association between third molar agenesis and dental maturity. Although some studies suggest that socioeconomic and nutritional factors may influence the prevalence of third molar agenesis [27, 28], dental age remains a reliable indicator for age estimation, since tooth development is highly stable and relatively unaffected by environmental factors [20, 29]. Theories have also proposed the existence of morphogenetic fields within tooth development, which may account for dental variability [30, 31]. From this perspective, the association between third molar agenesis and delayed dental maturation might reflect broader developmental patterns rather than being solely influenced by external conditions. Additionally, the sample represents only a specific geographic population, which may limit the generalizability of the findings. Future research should include broader, multicenter populations to validate and expand upon these results, considering potential differences in agenesis frequency and dental maturity across regions.

## Conclusions

Individuals with third molar agenesis exhibited delayed dental maturity in comparison with children with all four molars. The delay was more pronounced in patients with agenesis of three or four third molars, indicating that the extent of agenesis is associated with the magnitude of maturational delay. These findings suggest that third molar agenesis may serve as a developmental marker in clinical and forensic contexts. Nevertheless, the results should be interpreted with caution, as the study was based on a specific population and did not include variables such as genetic, nutritional, and socioeconomic factors. Broader, multicenter studies are warranted to validate and generalize these observations.

## Key points

1. Third molar agenesis is associated with delayed dental maturity measured by Demirjian’s method in Brazilian children.
2. The extent of dental delay increases with the number of missing third molars: patients missing 3 or 4 third molars showed significantly greater delays than those missing fewer or none.
3. Dental maturity assessments may benefit from considering third molar agenesis as a developmental marker in clinical and forensic contexts.
4. Variables such as socioeconomic status, nutrition, and genetic background were not evaluated, and the lack of external validation may limit the generalizability of the findings. Moreover, multicenter validation is needed to strengthen the conclusions.

## Data Availability

The data that support the findings of this study are available from the corresponding author upon reasonable request.
